# Supplementary material for: A New Subform? Fast-Progressing, Severe Neurological Deterioration Caused by Spinal Epidural Lipomatosis
Source: J Clin Med. 2022 Jan 12;11(2):366. doi: 10.3390/jcm11020366 (PMC8781155; doi:10.3390/jcm11020366)
Supplement: Supplementary file 1 [file jcm-11-00366-s001.zip › jcm-1528849-Figure S1.pdf]

1) <u>Pubmed search (November 7th, 2021)</u>

"((((lipomatosis[MeSH Terms]) AND "Spinal") AND "english"[Language]) AND "journal article"[Publication Type]) AND (acute OR progressive OR quick OR rapid* OR fast)"*

Studies identified: 43

2) <u>Scopus search (November 7th, 2021)</u>

"ALL ("Spinal Epidural Lipoma" OR "Spinal Lipoma*") AND ALL (quick* OR fast OR rapid* OR progressive OR acute) AND (LIMIT-TO (SRCTYPE, "j")) AND (LIMIT-TO (LANGUAGE, "English"))"*

Studies identified: 527

3) <u>Web of Science search (November 7th, 2021)</u>

"((ALL=((Spinal Epidural Lipoma OR Spinal Lipoma*) AND (Acute OR Rapid* OR Quick* OR Progressive)))) AND* ***LANGUAGE:*** *(English) AND* ***DOCUMENT TYPES:*** *(Article)"*

Studies identified: 138

4) <u>Cochrane Library search (November 7th, 2021)</u>

"("Epidural lipomatosis" OR "Spinal Lipoma") AND (Progressive OR Acute OR fast OR quick* OR rapid*))"*

Studies identified: 3

Figure legend:
Search terms and operators used for the four different search engines (PubMed, Scopus, Web of Science and Cochrane Library).

Figure S1. Systematic literature search terms.
