# Supplementary material for: A New Subform? Fast-Progressing, Severe Neurological Deterioration Caused by Spinal Epidural Lipomatosis
Source: J Clin Med. 2022 Jan 12;11(2):366. doi: 10.3390/jcm11020366 (PMC8781155; doi:10.3390/jcm11020366)
Supplement: Supplementary file 1 [file jcm-11-00366-s001.zip › jcm-1528849-Table S1.pdf]

Table S1. Quality assessment score used to rate the identified studies (cp. Table 1).

Parameter	Description	Value
Minimizing selection bias*		
Study design<br>Study source	Retrospective non-consecutive series / case reports	2
Minimizing information bias		
Year reported	No	0
	Yes	2
Baseline characteristics†	No	0
	Incomplete demographic and clinical data	2
	Complete demographic and clinical data	4
Primary diagnostic modality	Unclear	0
	CT/myelography	2
	MRI	4
SEL characteristics	Unclear/none	0
	Incomplete	2
	Spinal levels involved and maximum obstruction of the spinal canal reported	4
SEL-related data	Not reported	0
	(Putative) SEL risk factors were reported	4
Clinical symptoms and treatment	Not reported	0
	Incomplete	2
	Both were reported	4
Time	Descriptive (e.g., "fast" or "quick")	0
	Time from symptoms to diagnosis or treatment reported	2
	Both time points were reported	4
Histological confirmation	No / not reported	0
	Macroscopical confirmation of fat tissue	2
	Histological confirmation of SEL	4
Outcome	Not reported	0
	Short follow-up (≤ 6 months)	2
	Intermediate to long follow-up (> 6 months)	4

* No other studies than case reports and series were identified, limiting the score for "minimizing selection bias" to 2 out of a maximum of 8 points.
† All identified studies were small case series or case reports. Therefore, the entirety of the data was assumed as long as no contradiction was observed.
Abbreviation: SEL – spinal epidural lipomatosis.
