# Supplementary material for: A New Subform? Fast-Progressing, Severe Neurological Deterioration Caused by Spinal Epidural Lipomatosis
Source: J Clin Med. 2022 Jan 12;11(2):366. doi: 10.3390/jcm11020366 (PMC8781155; doi:10.3390/jcm11020366)
Supplement: Supplementary file 1 [file jcm-11-00366-s001.zip › jcm-1528849-Table S2.pdf]

Table S2: Detailed description of the neurological symptoms of the patients identified by the systematic literature review

ID	Sex	Age	Neurological deficits	Reference	Year
1	M	45	Severe paraparesis (2-3/5), loss of ambulation, sub-T10 hypoesthesia	Toshniwal et al.	1987
2	F	62	Rapidly progressive paraparesis (rapidly worsened to paraplegia with urinary retention)	Buthiau et al.	1988
3	M	52	Paraplegia, painful flexor spasms, hyperreflexia of both legs, bilateral positive Babinski's sign, sub-T7 hypoesthesia	Kaplan et al.	1989
4	M	20	Paraplegia and sub-T6 anesthesia with urine and fecal incontinence	Meisheri et al.	1996
5	M	27	3/5 paraparesis, reduced reflexes, urinary incontinence	Resnick et al.	2004
6	M	41	At admission: unsteady gait, sensory deficits, paraparesis;<br>12h after admission: paraplegia, urinary incontinence	Vince et al.	2005
7	M	60	Urinary and fecal incontinence and spastic paraparesis, impairment of tactile and pain sensation	Oikonomou et al.	2007
8	M	55	2/5 paraparesis, urinary retention, sub-T7 anesthesia	Lopez-Gonzalez et al.	2008
9	M	49	Paraplegia, areflexia of both legs, bilateral positive Babinski's sign, sub-T6 hypoesthesia, absent anal sphincter tone	Birmingham et al.	2009
10	F	35	Right side dominant paraparesis with inability to ambulate or stand	Stephenson et al.	2014
11	M	69	Acute incomplete cauda equina syndrome and bilateral foot drop	Tardivo et al.	2021
