# Supplementary material for: A New Subform? Fast-Progressing, Severe Neurological Deterioration Caused by Spinal Epidural Lipomatosis
Source: J Clin Med. 2022 Jan 12;11(2):366. doi: 10.3390/jcm11020366 (PMC8781155; doi:10.3390/jcm11020366)
Supplement: Supplementary file 1 [file jcm-11-00366-s001.zip › jcm-1528849-Text Case and Figure S2.pdf]

Journal of Clinical Medicine

Article

Supplementary Text – Case presentation:

A new subform? – Fast-progressing, severe neurological deterioration caused by spinal epidural lipomatosis

Thiemo Florin Dinger[1*], Maija Susanna Eerikäinen[2], Anna Michel[1], Oliver Gembruch[1], Marvin Darkwah Oppong[1], Mehdi Chihi[1], Tobias Blau[3], Anne-Kathrin Uerschels[1], Daniela Pierscianek[1], Cornelius Deuschl[2], Ramazan Jabbarli[1], Ulrich Sure[1] and Karsten Henning Wrede[1]

[1] University Hospital of Essen, Department of Neurosurgery and Spine Surgery, University of Duisburg-Essen; (Essen, North Rhine-Westphalia, Germany);
[2] University Hospital Essen, Institute for Diagnostic and Interventional Radiology and Neuroradiology, University of Duisburg-Essen (Essen, North Rhine-Westphalia, Germany);
[3] University Hospital of Essen, Institute of Neuropathology, University of Duisburg-Essen (Essen, North Rhine-Westphalia, Germany)

* Correspondence: Thiemo-Florin.Dinger@uk-essen.de; Tel.: 0049/ 201 723 1201

Citation: Lastname, F.; Lastname, F.; Lastname, F. Title. *J. Clin. Med.* **2022**, *10*, 366. https://doi.org/10.3390/jcm11020366

Academic Editors: Arash Moghaddam and Raban Heller

Received: 12 December 2021
Accepted: 10 January 2022
Published: 12 January 2022

Publisher's Note: MDPI stays neutral with regard to jurisdictional claims in published maps and institutional affiliations.

Copyright: © 2022 by the authors. Submitted for possible open access publication under the terms and conditions of the Creative Commons Attribution (CC BY) license (https://creativecommons.org/licenses/by/4.0/).

1. Supplementary Materials and Methods

1.1. Case report

The presentation of the current case was performed according to the case report guidelines “CARE”[1]. Informed consent was obtained.

2. Supplementary Results

2.1. Case

A 67 years old Caucasian male was presented to the neurosurgical department with a rapidly progressing neurological deterioration (<36 h from first symptoms to functional paraplegia). He suffered from severe paraparesis, parahypesthesia sub-T5, and urinary and anal incontinence. The patient's medical history revealed an advanced pulmonary adenocarcinoma with right frontal metastasis treated by radiotherapy. During treatment, the patient intermittently received corticosteroids for 16 months. Additionally, the medical history included diabetes, alcohol dependency (1L beer/day), adiposity, hepatic steatosis along with tobacco consumption (40 pack-years), and labyrinthine hearing impairment (**Figure S2**).

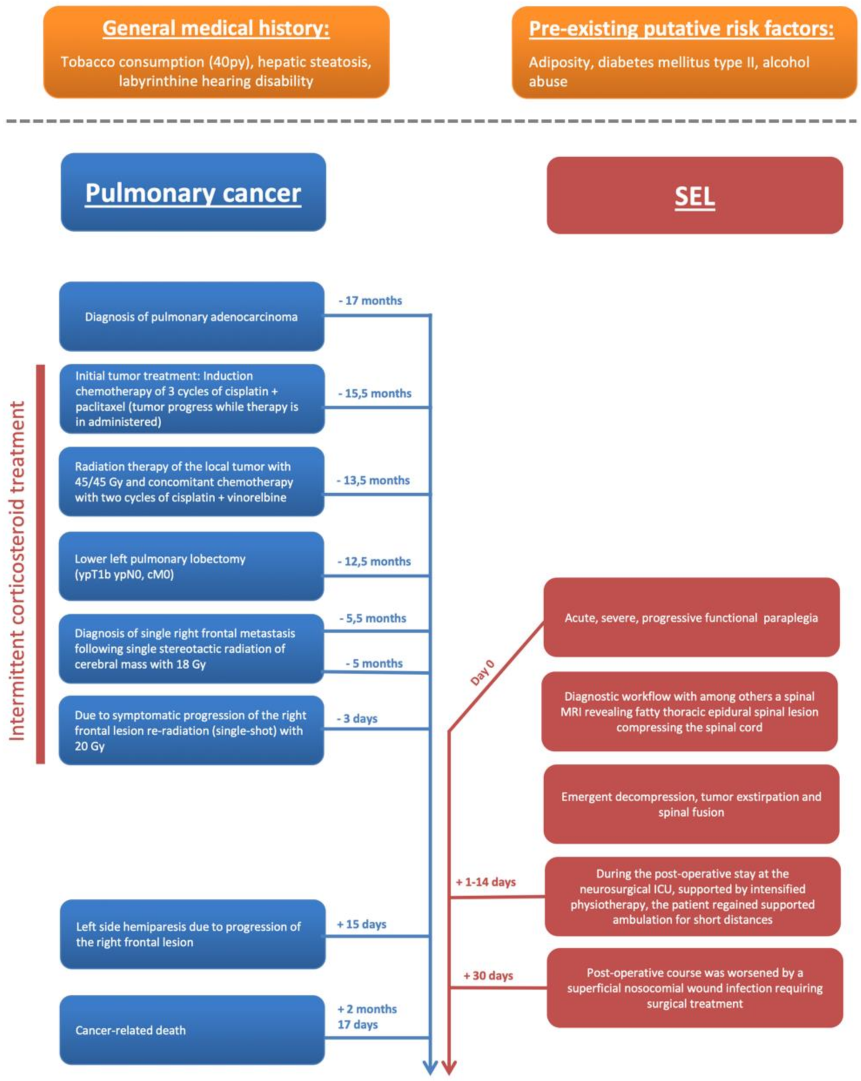

Figure S2. Timeline summary of the presented case. Timeline of the current case in chronological relation to the acute, functional paraplegia. Pre-existing diseases are listed above the dashed line separated in those probably unrelated and related (putative risk factors) to SEL. Below the dashed line, the events are separately listed for pulmonary cancer (blue) and SEL (red).

An emergent spinal MRI revealed an epidural fat isointense lesion extending from T2 to T10. The mass severely compressed the spinal cord, causing myelopathy (**Figure 3C** and **Figure S2** for quantification). Retrospectively, SEL could already be seen in the PET/CT scan at the time of the diagnosis of lung cancer (**Figure 3A**). However, SEL showed a significant relative growth of 12% over 17 months (**Figure 3A-C & Figure S2**).

We performed an emergent laminectomy from T3-T9 with craniocaudal extension and removal of a large epidural fat layer (**Figure 4A** & **Video S1**). Due to spinal instability caused by multiple-level decompression, the patient required spinal fixation from T3-T9 (**Figure 4B**). Postoperative CT scan confirmed good decompression of the spinal cord and correct implant position (**Figure 4C**).

From postoperative day one, the patient made good progress under intensified physiotherapy. He regained a 4-/5 muscle strength (Medical Research Council Manual Muscle Testing scale) and ambulated with a supporting physiotherapist after seven days of training.

Neuropathologists confirmed the diagnosis of SEL with histological findings of benign hypertrophic unencapsulated fat cells (**Figure 4D**).

On the 30th postoperative day, the patient had to undergo surgical debridement for a local superficial wound infection.

On day 77, the patient died due to a decreasing general state of health caused by a progress of his malignant disease's.

Informed Consent Statement: Written informed consent for publication has been obtained from the patient to publish this paper.

References

1. Riley, D.S.; Barber, M.S.; Kienle, G.S.; Aronson, J.; Schoen-Angerer, T. von; Tugwell, P.; Kiene, H.; Helfand, M.; Altman, D.G.; Sox, H.; et al. CARE 2013 Explanations and Elaborations: Reporting Guidelines for Case Reports. *J Clin Epidemiol* 2017, *89*, 218–235, doi:10.1016/j.jclinepi.2017.04.026.
